# Visual Outcome Following Manual Small Incision Cataract Surgery at a Tertiary Center in South India

**DOI:** 10.7759/cureus.20687

**Published:** 2021-12-25

**Authors:** Chethana Warad, Arvind Tenagi, Pranitha Satarasi, Dhruv Goyal, Riya Mendpara, Umesh Harakuni, Shivanand C Bubanale, Smitha K S, Bhagyajyothi B K, Vivek Wani

**Affiliations:** 1 Department of Ophthalmology, Jawaharlal Nehru Medical College, Belagavi, IND

**Keywords:** senile cataract, blindness prevention, visual outcome, best corrected visual acuity, manual small incision cataract surgery

## Abstract

Introduction

Cataract being one of the leading causes of avertible blindness has been found to be quite prevalent in developing countries like India. The National Programme for Control of Blindness (NPCB) aims at reducing blindness due to cataract via cataract control programs. The most commonly performed surgery is the manual small incision cataract surgery (MSICS).

Aim

The aim of this study was to determine the visual acuity and outcome in patients who underwent MSICS in a tertiary hospital in south India.

Methodology

A prospective longitudinal interventional study was conducted in a tertiary hospital in Karnataka, India, over a period of nine months. A total of 105 eyes underwent MSICS and were followed up for one month to assess their postoperative visual outcome and complications, if any. During this period, they were started on antibiotic + steroid combination eyedrops, which were tapered over four weeks.

Results

Results were assessed based on visual grading categorized by the World Health Organization (WHO). A total of 103 (98.1%) patients had good vision, i.e., visual acuity of 6/6 - 6/18, followed by two (1.9%) who had moderate vision i.e., visual acuity of <6/18 - 3/60, and none were blind or with visual acuity of <3/60. Complications seen intraoperatively in two patients (1.9%) were iris prolapse and posterior capsular rent, respectively, and postoperatively one (0.95%) patient showed hyphema with inferior haptic in the anterior chamber.

Conclusion

This study proves that a good visual outcome with a low complication rate can be achieved after MSICS with posterior chamber intraocular lens implantation.

## Introduction

Cataract is one of the leading causes of avertible blindness, contributing to 66.2% of the estimated 50 million cases of preventable blindness [1]. Blindness is appreciably severe in developing countries like India due to ignorance, poverty, and the dearth of medical resources in the peripheral areas [1,2].

The National Programme for Control of Blindness (NPCB) via cataract control programmes aims at clearing the backlog of blindness due to cataract [3]. These programmes are increasing the number of surgical facilities dispensed, most commonly manual small incision cataract surgery (MSICS), which is a technique that can be employed in suboptimal conditions and incurs a low maintenance cost as compared to phacoemulsification [4]. MSICS takes less time to perform than phacoemulsification and is more cost-effective and the most appropriate method to be performed in developing countries for high-volume cataract surgeries [5].

However, the outcomes of MSICS and the improvement in visual acuity of patients post-surgery are not always as anticipated, and more attention needs to be paid to the surgical amenities being provided [6].

The aim of our study is to determine the visual acuity and outcome in patients who underwent MSICS in a tertiary hospital in south India.

## Materials and methods

JNMC Institutional Ethics Committee on Human Subjects Research approved the study (MDC/DOME/220).

A prospective longitudinal interventional study was conducted in a tertiary hospital in Karnataka over a period of nine months from January 1, 2021, to August 30, 2021. All the patients above the age of 40 years having diminution of vision due to cataract who underwent cataract surgery by MSICS were included in the study.

Patients below the age of 40 years and those with other causes of diminution of vision, such glaucoma, diabetic retinopathy, corneal opacities, any macular pathology, and traumatic cataract, were excluded from the study.

After taking a brief history, visual acuity, intraocular pressure by non-contact tonometer, lacrimal sac syringing, blood pressure, random blood sugar, and physician review for fitness, fundus examination was conducted for all the patients. Xylocaine sensitivity test, COVID-19 rapid antigen test, HIV, and HbsAg serology was performed for all patients. Antibiotic eyedrops were instilled one day prior to the surgery. Tropicamide eyedrops were used to dilate the pupil. All the surgeries were performed by a single surgeon after taking informed and written consent for the surgery.

In terms of operative procedure, under all aseptic precaution, peribulbar block was given. Universal eye speculum was put. Superior rectus bridle suture was taken with 4-0 silk suture. Fornix-based conjunctival flap was raised. Hemostasis was achieved by wet field cautery. Straight scleral incision was made at 2 mm away from the limbus. A tunnel was made with a crescent. Anterior chamber (AC) entry was done with keratome. Anterior capsulotomy was performed. Hydrodissection was performed, and the nucleus was prolapsed into the AC and delivered by phacosandwich technique. Cortical matter was aspirated followed by posterior chamber intraocular lens (PCIOL) placement in the capsular bag. Thorough AC wash was done. Subconjunctival dexamethasone + gentamycin injection was given.

Postoperatively, oral antibiotics were given for three days. Analgesics were given if needed. Antibiotic + steroid combination eyedrops were tapered over four weeks. Tropicamide eyedrops were given to put once at night for one week. Postoperatively, the visual acuity was assessed on day 1, first week, and fourth week.

Cataract is defined as the clouding of the crystalline intraocular lens occluding the rays entering the eye and partially or completely impeding the red reflex on distant direct ophthalmoscopy causing visual impairment [7]. 7Visual outcome post-MSICS was categorized by the World Health Organization (WHO) as follows [1]:

· Normal to mild visual impairment: 6-6 - 6/18

· Moderate visual impairment: 6/18 - 6/60

· Severe visual impairment: <6/60 - >3/60

· Blind: <3/60

Data were analyzed using statistical software R Version 4.1.1 (R Foundation for Statistical Computing, Vienna, Austria) and Microsoft Excel (Microsoft, Redmond, WA). Continuous variables were represented as mean ± SD, and categorical variables were represented as frequency. The McNemar test was used to compare the pre- and postoperative vision. P-value less than or equal to 0.05 indicated statistical significance.

## Results

The staging of cataract, as shown in Table 1, was such that majority, i.e., 90 (85.71%) were immature cataracts and 15 (14.29%) were mature cataracts. Table 2 shows the same. A total of 105 eyes of 94 patients who met the inclusion criteria were included in the study. Age of the patient ranged from 40 to 80 years, with a mean age of 62.37 ± 9.01 years. Of the patients, 56 (59.57%) were females and 38 (40.43%) were males.

**Table 1:** Staging of cataract

Staging	Number of eyes (%)
Immature	90 (85.71%)
Mature	15 (14.29%)

**Table 2:** Distribution of age and gender in the study

Demographic data		Number of patients (%)
Age (in years)	40-49	10 (10.64%)
	50-59	17 (18.09%)
	60-69	45 (47.87%)
	70-79	21 (22.34%)
	80-89	1 (1.06%)
Gender	Female	56 (59.57%)
	Male	38 (40.43%)

Table 3 shows the pre- and postoperative visual acuity.

**Table 3:** Pre- and postoperative visual acuity MSICS, manual small incision cataract surgery

		No. of patients	
		Before MSICS	After four weeks of MSICS
Visual Impairment	Blind (<3/60)	48 (45.71%)	0(0%)
	Moderate vision (<6/18 - 3/60)	48 (45.71%)	2 (1.9%)
	Good vision (6/6 - 6/18)	9 (8.57%)	103 (98.1%)

Among 105 operated eyes, the visual acuity showed that 103 (98.1%) patients had good vision, i.e., visual acuity of 6/6 - 6/18, followed by two (1.9%) patients who had moderate vision, i.e., visual acuity of <6/18 - 3/60, and none were blind or with a visual acuity of <3/60.

Using the McNemar test, there was significant change in the distribution of vision before and after surgery.

Intraoperatively, two patients (1.9%) incurred complications of iris prolapse and posterior capsular rent, respectively. The iris prolapse was managed by repositioning the iris and suturing the section, whereas the posterior capsular rent was managed by placing the PCIOL in the sulcus.

One (0.95%) patient showed grade one hyphema with inferior haptic in AC on postoperative day 1. The hyphema resolved over the following week. A total of 102 (98.07%) eyes showed no intaoperative or postoperative complications. Table 4 shows the complication statistics.

**Table 4:** Complications statistics AC, anterior chamber

Intraoperative complications	Number of eyes (%)
Posterior capsule rent	1 (0.95%)
Iris prolapse	1 (0.95%)
Postoperative complications	Number of eyes (%)
Inferior haptic in the AC	1 (0.95%)
Nil	102 (98.07%)

## Discussion

This was a prospective longitudinal study conducted in a tertiary hospital in South India assessing the visual outcomes and complications over four weeks in patients who had undergone MSICS.

The age distribution was found to be such that majority were in the range of 60-69 years, with a mean age of 62.37 ± 9.01 years. This is very similar to previous studies. Wetarini et al. [4] found 43.5% of the cataract patients in this age group, with a mean age of 63 ± 10 years in Bali, Indonesia. Nwosu and Onyekwe [8] in Nigeria showed comparable findings of a mean age of 64 years, while studies conducted in India also showed the same. Khandekar et al. conducted a study in central India and found 41.86 % to be in the age group of 61-70 years [9].

More women (59.57%) were found to have cataract in our study. It has been proven in prior studies that women have a more age-adjusted risk of developing cataract than men. This could be due to longer average lifespan of women and thus having a higher prevalence of undergoing cataract surgery [9-12].

Cataract is one of the leading causes of blindness in India and the world. The presenting visual acuity is parallel to other studies [13]. Most studies show that in developing countries, majority of patients present to the hospital at a stage at which they are blind or almost blind in at least one eye [14-16], as opposed to developed countries where patients present earlier, with a better visual acuity. In a study conducted in Sub-Saharan Africa, the predominant (36.9%) visual acuity was hand motion close to face [14]. A study conducted in Nepal concluded that one in eight patients of the sample population operated for cataract were blind at presentation [16].

Cataract surgeries are the one and only available solution to this issue, and MSICS performed in this study has shown favorable visual outcome, i.e., among 105 operated eyes, the visual acuity showed that 103 (98.1%) patients had good vision, i.e., visual acuity of 6/6 - 6/18, followed by two (1.9%) patients who had moderate vision. Other studies revealed similar favorable results: 87% in a study by Khandekar et al. [9] and 88% and 99% in two separate studies by Venkatesh et al. [17,18]. Our study has shown better results than studies conducted in South-western Nigeria [19] and Kenya [13], with 54.1% and 77.1%-89.4% having good vision, respectively, post-small incision cataract surgery.

According to the WHO and the International Agency for the Prevention of Blindness (IAPB) action plan, >85% should have a good vision of 6/6 - 6/18 post-cataract surgery [20]. Our study has exceeded this target at a four-week follow-up period

Assessing visual acuity post-cataract surgery is a routine practice for surgical evaluation. Various factors influence the visual outcome, such as stage of cataract, ocular and systemic comorbidities, surgical technique, surgical skills, and complications during surgery [21-23].

Our study had fewer overall complications (2.86%) such as posterior capsular rent, iris prolapse, and hyphema, as compared to other studies that revealed higher number complications [9,19,24].

The limitation of our study is its small sample size of 105 eyes and a short follow-up duration of one month postoperatively. Thus, a much larger sample size with a longer observation period is ideally required for assessing visual outcomes with accuracy post-MSICS.

## Conclusions

This study conducted at a tertiary hospital in South India proves that a good visual outcome with a low complication rate can be achieved after MSICS with PCIOL implantation. Cataract surgeries are the one and only available solution, and MSICS performed in this study has shown favorable visual outcome.

It is an easily available, affordable technique that can be used safely and can be dispensed at a large scale in developing countries. This study proves that MSICS can be used as an effective and affordable treatment option especially in sections of the country that lack advanced resources. It is an excellent tool to eliminate preventable blindness due to cataract.
